# Adaptation of Commensal *Escherichia coli* in Tomato Fruits: Motility, Stress, Virulence

**DOI:** 10.3390/biology12040633

**Published:** 2023-04-21

**Authors:** Alberto Vassallo, Roberta Amoriello, Prandvera Guri, Lorenzo Casbarra, Matteo Ramazzotti, Marco Zaccaroni, Clara Ballerini, Duccio Cavalieri, Massimiliano Marvasi

**Affiliations:** 1 Department of Biology, University of Florence, 50019 Sesto Fiorentino, Italy; alberto.vassallo@unicam.it (A.V.); pranvdera.guri@stud.unifi.it (P.G.); duccio.cavalieri@unifi.it (D.C.); 2 Department of Biomedical, Experimental and Clinical Sciences, University of Florence, 50134 Florence, Italy; roberta.amoriello@unifi.it (R.A.); clara.ballerini@unifi.it (C.B.); 3 Department of Experimental and Clinical Biomedical Sciences, University of Florence, 50134 Florence, Italy; lorenzo.casbarra@unifi.it (L.C.); matteo.ramazzotti@unifi.it (M.R.)

**Keywords:** bacterial adaptation, fruit contamination, food safety, *Lycopersicum esculentum*, dendritic cells

## Abstract

**Simple Summary:**

When pathogenic bacteria contaminate food, they can harm humans, causing, for example, gastrointestinal disorders. This is a main concern, especially in the case of foods that are consumed fresh and/or are not cooked following common hygiene standards. Thus, it is important to understand how bacteria can survive and prosper once they have contaminated fruits and vegetables, regardless of the presence of defense mechanisms in these plant tissues. In this work, we investigated the ability of a harmless *E. coli* strain’s ability to adapt to a tomato’s external portion, where bacteria encounter harsh growth conditions. *E. coli* grown in tomatoes was isolated and compared with *E. coli* grown in standard laboratory conditions: this comparison allowed us to identify potential molecular determinants helping *E. coli* to survive in tomatoes. This investigation was conducted through sequencing of DNA and through assays proving that growth in tomatoes altered, at least in part, some features of this bacterium, such as its ability to withstand chemical compounds.

**Abstract:**

Food contamination can be a serious concern for public health because it can be related to the severe spreading of pathogens. This is a main issue, especially in the case of fresh fruits and vegetables; indeed, they have often been associated with gastrointestinal outbreak events, due to contamination with pathogenic bacteria. However, little is known about the physiological adaptation and bacterial response to stresses encountered in the host plant. Thus, this work aimed to investigate the adaptation of a commensal *E. coli* strain while growing in tomato pericarp. Pre-adapted and non-adapted cells were compared and used to contaminate tomatoes, demonstrating that pre-adaptation boosted cell proliferation. DNA extracted from pre-adapted and non-adapted cells was sequenced, and their methylation profiles were compared. Hence, genes involved in cell adhesion and resistance against toxic compounds were identified as genes involved in adaptation, and their expression was compared in these two experimental conditions. Finally, pre-adapted and non-adapted *E. coli* were tested for their ability to resist the presence of toxic compounds, demonstrating that adaptation exerted a protective effect. In conclusion, this work provides new information about the physiological adaptation of bacteria colonizing the tomato fruit pericarp.

## 1. Introduction

Members of the Enterobacteriaceae can adapt to a wide variety of environments [1], including plants. *Escherichia coli,* for example, is a widespread gut commensal and often a versatile pathogen of public health concern [2]. Plant contamination with human pathogens has important implications for human health, as vegetables are increasingly recognized as vehicles of *Salmonella enterica*, pathogenic *E. coli*, and other pathogens [3,4,5]. Outbreaks of gastroenteritis caused by pathogenic *E. coli* strains [4] and related to the consumption of fresh fruit and vegetables show that this human pathogen can contaminate products at any stage of the production cycle [6].

Bacteria can penetrate plant tissue through wounds, and Enterobacteria such as *E. coli* can duplicate in plants. In the case of tomato fruits, bacteria can penetrate the fruit’s pericarp area and specifically replicate within the intercellular spaces [7]. Nevertheless, only minor information is available about the ecology and adaptation to the host of *E. coli* in tomato pericarp and, in general, in the plant. In the pericarp tissue, bacteria find a complex environment. Indeed, for example, the production of H_2_O_2_ is plentiful in plant tissues, as reported in the case of the intercellular spaces of tomatoes and apples [8,9] and the fruits of *Capsicum annuum* (bell pepper), which exhibit microbursts of H_2_O_2_ when facing particular stressors such as invading pathogens [10].

Recent research has shown that *E. coli* isolated from colonized leaves could progressively colonize lettuce seedlings to higher titers, suggesting the presence of a fast adaptation process [11]. Moreover, these colonizing *E. coli* cells isolated from leaves also showed a dramatic rise in tolerance to oxidative stress [11].

Limited information is also available on how this adaptation to the plant environment relates to the stimulation of *E. coli* during infection in human hosts. For instance, *E. coli* O157:H7 (EHEC) present on the surface of living radish sprouts showed limited activation of the pathogenicity islands, while the highest transcriptional activity of these genes was observed when *E. coli* was cultivated in Lysogeny Broth (LB) medium, minimal medium, and after treatment with antibiotics [12]. Lower gene activation in *E. coli* while persisting in *Arabidopsis* was also detected in further experiments [13]. It is possible that the progressive adaptation in the plant environment would strengthen the bacterial stress responses.

In this work, we aimed to furtherly study the interaction of a commensal strain of *E. coli* and tomato fruit, as tomato represents a major commodity for the food industry worldwide [3]. This interaction was investigated by analyzing epigenetic modifications that occurred in the bacterial DNA after growth in tomatoes. In particular, DNA methylation was studied because it is the main epigenetic regulation controlling gene expression in bacteria [14]. Indeed, DNA methylation can control protein binding to target DNA, DNA curvature, and gene transcription, besides being involved in well-studied molecular mechanisms such as chromosome replication, DNA damage repair, defense against restriction enzyme-mediated cleavages, and phase variation [15]. Thus, this work took advantage of third-generation DNA sequencing methods, as they can directly identify chemical modifications of nucleobases; in this case, DNA was sequenced by nanopore technology that can identify modified bases by measuring changes in the electric current while DNA passes through protein pores [16,17].

These results showed that adaptation induced differential nucleotide methylations, leading to reduced cell motility and increased resistance against stress. Moreover, pre-adaptation in tomato fruits fostered elicitation of inflammatory stimuli in human dendritic cells. Other studies have shown that some genotypes of commensal *E. coli*, isolated from healthy conventional microbiota mice and representing distinct populations of *E. coli*, elicited strain-specific disease phenotypes and immunopathological changes following treatment with the inflammatory stimulus [18]. This is interesting since, in general, commensal anaerobic gut bacteria can attenuate inflammation [19].

## 2. Materials and Methods

### 2.1. Bacterial Strain and Plant Material

The strain *Escherichia coli* ATCC 35218 was used in this study. Although *E. coli* ATCC 35218 is classified as a Biosafety Level 1 strain, it holds specific virulence genes shared with different *E. coli* pathotypes causing human and animal infections, such as *fimH*, *papA*, *papC*, *papG*, *papE*, *sfaS*, *hlyA* (gene for α-hemolysin), *kpsM*, *fyuA*, and *ompT* [20]. The strain also hosts a *bla* gene conferring resistance to ampicillin. Tomato fruit cultivar ‘SF’ (*Lycopersicum esculentum*) was used for the experiments and purchased at the local grocery store. Only tomatoes at the “ripe” stage 6 were used as indicated by the USDA Color Classification Requirements. Fruits were first rinsed with tap water, then with deionized water, and finally wiped with paper. Fruits were not subjected to any further decontamination procedure of the external surface to mimic a realistic day life situation. The quality and integrity of tomatoes were visually checked before and during the incubation with *E. coli*. All fruits that showed, at any stage of the experiments, any damage or sign of spoilage were discarded.

### 2.2. Tomato Fruits Contamination

Tomato pericarp contamination was performed as previously described [5]. Briefly, *E. coli* was grown overnight at 37 °C in Lysogeny Broth (LB) (Fisher Scientific) under shaking (200 rpm). One mL of culture was pelleted, washed three times in PBS (pH 7.0), and diluted in sterile water. Three μL of the suspension (containing a final concentration of about 10^2^ colony-forming units (CFU)) were spotted onto three shallow wounds (~1 mm) under the fruit epidermis. Contaminated fruits were incubated at room temperature for three days. Some of the tomatoes were used for the growth curve: upon completion of the incubation, tomatoes were homogenized in an equal volume of PBS (pH 7.0) (Fisher Scientific) using a stomacher (Sevard) (200 rpm for 1 min), and the suspensions were plated onto MacConkey Agar (Oxoid) plates and incubated at 37 °C overnight. Proliferation was calculated by dividing the total CFU recovered from each tomato by the total CFU inoculated into each fruit. This accounted for differences in tomato sizes. The ratios were further subjected to the log10 transformation. A total of 14 tomatoes were used for the growth curve, while 11 or 12 tomato fruits were used for the adaptation experiments. All fruits were purchased on at least two different days.

Alongside the contamination experiments, control tomato fruits were used to exclude the presence of prior *E. coli* contamination. In each experiment, these control fruits were tested onto MacConkey Agar plates using swabs from their surface and homogenate (obtained with the stomacher) to detect colonies morphologically similar to those of *E. coli* ATCC 35218. No *E. coli* colonies were detected in these controls. Other bacteria present on the external surface of fruits were not monitored. Moreover, presence of other putative ampicillin-resistant bacteria than *E. coli* ATCC 35218 was monitored and excluded by checking the presence of beta-lactamase resistance genes in the DNA used for the differential methylation analysis (see Section 2.3 for further details).

### 2.3. Differential Methylation Analysis

#### 2.3.1. Tomato Contamination and *E. coli* ^TOM^ Isolation

*E. coli* were grown overnight at 37 °C in Lysogeny Broth (LB) (Fisher Scientific) under shaking (180 rpm). One mL of the overnight grown culture was washed twice in PBS (pH 7.0) and finally resuspended in 1 mL of sterile PBS (pH 7.0). The titer of this suspension was determined by counting CFU on MacConkey Agar plates after overnight incubation at 37 °C.

Five hundred μL of washed cells were inoculated in 50 mL of pre-heated LB medium in a 250 mL Erlenmeyer flask. This culture was incubated at 37 °C under shaking (180 rpm) and stopped when the exponential phase was reached. The culture was centrifuged, and the pellet was collected and stored at −20 °C until use: this bacterial pellet (indicated hereinafter as *E. coli* ^LB^) was used as reference to determine the differential nucleotide methylation profile.

Suspension of the *E. coli* washed cells was also diluted in PBS, and 3 μL of the 10^−3^ dilution were spotted into shallow (~1 mm) wounds in tomato epidermis made with a sterile micropipette tip. Each tomato had eight wounds, and twelve tomatoes were used for each experiment: this number of wounds allowed to maximize the yield of *E. coli* cells used for the differential DNA methylation analysis without compromising fruit integrity. The number of *E. coli* cells inoculated in each wound was determined by counting CFU on MacConkey Agar plates after overnight incubation at 37 °C: ~10^3^ cells were inoculated in each wound. Contaminated tomatoes were incubated at room temperature for two days. Bacterial cells were harvested from tomatoes using the following protocol: plugs (~1 × 1 × 0.1 cm) centered on the wound used for contamination were collected in 200 mL of sterile PBS in a sterile stomacher plastic bag. Plugs were macerated using a stomacher (Sevard) with four rounds of 230 rpm for 30 s. Stomached tomatoes were filtered with a tea colander to remove large particulate, then with Whatmann n.8 filter paper under vacuum. The filtered suspension was centrifuged at 7000× *g* for 5 min, and the supernatant was removed, leaving about 5 mL of it. The obtained pellet was resuspended gently without breaking its red portion formed by the debris of tomato, and the supernatant, containing bacterial cells, was transferred in a new centrifuge tube. This final suspension was centrifuged at 10,000× *g* for 3 min, and the white pellet obtained was resuspended in 200 μL of sterile PBS. The number of harvested cells (*E. coli* ^TOM^) was estimated by counting CFU on MacConkey Agar plates.

#### 2.3.2. DNA Extraction and Sequencing

Differential methylation sites were determined by comparing the methylation profiles of *E. coli* ^TOM^ and *E. coli* ^LB^ samples, with the latter used as a reference. DNA was extracted using the DNeasy PowerSoil Pro Kit (Qiagen, Hilden, Germany) and following the protocol provided by the manufacturer. DNA extracted from *E. coli* ^TOM^ and *E. coli* ^LB^ was sequenced using MinION (Oxford Nanopore Technology, Oxford, UK) and a PCR-free approach following the native barcoding genomic DNA protocol (version NBE_9065_v109_revY_14Aug2019), as previously described [21]. In brief, 1 μg of each sample DNA was repaired and end-prepped using the NEBNext Companion Module for Oxford Nanopore Technologies Ligation Sequencing (New England Biolabs, Ipswich, MA, USA). End-prepped DNA samples were barcoded using Native Barcoding Expansion 1–12 and NEB Blunt/TA Ligase Master Mix (New England Biolabs). After purification, equimolar amounts of barcoded DNA samples were pooled and ligated to adapters. DNA library was enriched with >3 kb-long fragments using the Long Fragment Buffer of the Ligation Sequencing Kit (Oxford Nanopore Technology). DNA library was immediately sequenced with an R9.4.1 Flow Cell (Oxford Nanopore Technology) and a MinION MK1B (Oxford Nanopore Technology). Methylation analysis was performed with Oxford Nanopore Technologies Tombo v.1.5.1 (nanoporetech.github.io/tombo/). Briefly, multi-read fast5 data were basecalled with guppy v.6.0.1 and demultiplexed with ont_fast5_api tool. Sample-specific fast5 files were resquiggled using the Tombo command tombo-resquiggle, using the fasta file of *Escherichia coli* ATCC 35218 from ATCC as reference sequence. Differential signals between LB growth and tomato growth were obtained with the Tombo detect_modifications command using the level_sample_compare mode using *t*-test as the main statistic and extracting -log10 *p*-values. For circular barplots, the Circos software [22] was used, plotting methylations of forward or reverse strand if *p*-value of differential methylation was lower than 0.001. The criteria to select differentially methylated C for further investigations were the following: (i) differences in methylation of Cs were in all three biological replicas of *E. coli* ^TOM^ versus *E. coli* ^LB^ and vice versa; (ii) at least 2 differentially methylated Cs were near each other within a 500 nucleotide-long interval. Once these two criteria were met, the expression of the genes having differentially methylated Cs was further investigated.

#### 2.3.3. qPCR Analysis

Total RNA was extracted from *E. coli* ^TOM^ and *E. coli* ^LB^ by using the RNeasy Mini Kit (Qiagen) according to the manufacturer’s instructions. The presence and integrity of RNA were checked by visualizing on 1.3% *w/v* agarose gel electrophoresis. Samples were quantified with Tecan Spectrophotometer according to manufacturer’s instructions. DNA was degraded with DNAse (Thermo Scientific), and RNeasy MinElute Cleanup Kit (Qiagen) was used to purify the DNA-free RNA. DNA-free RNA was tested via standard PCR amplification to ensure the complete removal of genomic DNA by using 16S rDNA primers (Table S1). cDNA synthesis was performed by using PrimeScript RT reagent Kit (Takara) according to the user manual by using random hexamer primers. qPCR was performed on a QuantStudio 7 Flex (Applied Biosystem, Waltham, MA, USA) instrument and using Powerup SYBR Green Master Mix (Applied Biosystem, Waltham, MA, USA) according to the user guide specifications. Negative control was carried out by using PCR-grade water instead of cDNA template. *E. coli* genes *narG*, *papA_1*, *fimH*, *atoB-C-D*, *acrF*, *murE-ftsl-ftsL*, *fliC*, and *flHD* were tested, whereas *rpoD* gene, coding for the RNA polymerase σ^70^ factor, was used as internal reference gene. qPCR was performed by using the following cycles: initial denaturation at 50 °C for 2 min, then 95 °C for 2 min, 40 cycles of denaturation at 95 °C for 15 sec, annealing at 59 °C for 15 sec, and extension at 72 °C for 30 sec. Primers used in PCR reactions are listed in Table S1. Minimum requirement tests to ensure specific amplifications were performed as recommended by the MIQE Guideline [23]. PCR amplification efficiency was established by means of calibration curves on all genes and melting curves to check single amplification. Three biological replicas and three technical replicas were used for each gene. Livak (2−ΔΔCt) method was used to analyze gene expression.

### 2.4. Growth Curves in the Presence of Antibiotics and H_2_O_2_

An overnight-grown culture of *E. coli* ATCC 35218 was diluted up to OD_600_ = 0.1 and aliquoted in 250 µL of LB medium in 96-well plate (Sarstedt, Nümbrecht, Germany) with different concentrations of antimicrobials: ampicillin at 0–100 µg/mL, nalidixic acid at 0–1–10 µg/mL, and H_2_O_2_ at 0–0.34–3.4 mM. Plates were incubated in a Tecan plate reader (Infinite 200 PRO, Tecan, Männedorf, Switzerland) at 37 °C in slow shaking for 12 h with OD_600_ measured every 30 min.

### 2.5. Motility Test

Motility was assessed with the method of MacConkey Agar to compare *E. coli* ^TOM^ and *E. coli* ^LB^. Briefly, MacConkey Agar (Oxoid) was diluted 1:4 and poured into a sterile tube. When solid, the medium was inoculated with overnight-grown *E. coli* tester cultures by inserting a sterile loop into the center of the medium. Color variation of the pH indicator in the medium was used to highlight bacterial spread because the medium turned yellow in the case of lactose fermentation.

### 2.6. Experiments with Human Monocyte-Derived Dendritic Cells

#### 2.6.1. Monocyte-Derived Dendritic Cells Differentiation

Human monocyte-derived dendritic cells (mDCs) were differentiated in vitro. Peripheral blood mononuclear cells (PBMCs) from 2 healthy donors were collected by density gradient centrifugation using Pancoll (density: 1.077 g/mL; Bioclass Srl, Pistoia, Italy) at 1500 rpm for 30 min, RT. From collected PBMCs, CD14+ monocytes were purified by immunomagnetic selection using the human CD14 MicroBeads kit (Miltenyi Biotec, Bergisch Gladbach, Germany) by elution of cell suspension through an LS column placed on a suitable Miltenyi MACS separator, following manufacturer’s protocol. Isolated CD14+ cells were washed once in MACS buffer (PBS 1X with 0.5% bovine serum albumin and 2 mM ethylenediaminetetraacetic acid) at 1500 rpm, RT, for 7 min, and then resuspended in complete RPMI 1640 medium (10% fetal bovine serum; 1% penicillin/streptomycin; 1% sodium pyruvate; 1% L-glutammine; 1% Hepes buffer) and counted. Monocytes were then plated at a density of 2 × 10^6^/mL in complete RPMI 1640 medium, at 37 °C and 5% CO_2_, for 7 days, in the presence of 100 ng/mL of granulocyte-macrophage colony-stimulating factor (GM-CSF; R&D Systems, Minneapolis, MI, USA) and 50 ng/mL of interleukin-4 (IL-4; R&D Systems, USA).

#### 2.6.2. Viability and Activation of *E. coli*-Infected Monocyte-Derived Dendritic Cells

After 7 days of differentiation, mDCs were washed once at 1300 rpm, 10 min, RT. Therefore, supernatant was completely discarded, and cells were resuspended in antibiotic-free incomplete RPMI 1640 medium, supplemented with only 10% fetal bovine serum. After being counted, mDCs were plated at a density of 5 × 10^5^ cells/500 μL in incomplete RPMI 1640 medium in the following conditions: (1) unstimulated; (2) stimulated with lipopolysaccharide (LPS); (3) stimulated with *E. coli* ^LB^ (10^6^ CFU/500 μL); (4) stimulated with *E. coli* ^TOM^ (10^6^ CFU/500 μL); and (5) in the presence of non-contaminated tomato, as control. After 2 h of infection, half of the medium (250 μL) from each well was discarded and replaced with 250 μL of complete RPMI 1640 medium, then 1 μg/mL of LPS was added to the appropriated wells, and cells were incubated overnight at 37 °C, 5% CO_2_. Afterward, mDCs were analyzed by CyFlow Space flow cytometer (Sysmex Partec, Görlitz, Germany) for viability, detecting necrotic cells by propidium iodide (PI; 2.5 μg/mL; Molecular Probes, Eugene, OR, USA), and for activation phenotype by labeling cells for 20 min, RT, in the dark, with the following fluorescent anti-human antibodies: HLA-DR PerCP (clone L243); CD80 FITC (clone 2D10.4); and CD86 PE (clone IT2.2), all from eBioscence (USA). Flow cytometry data were acquired by the FloMax software (Sysmex Partec, Germany).

Buffy coats were collected from 2 anonymous healthy donors at the Transfusion Unit at Careggi University Hospital in Florence, Italy. The utilization of donor material, not destined to diagnostic standard procedures and registered with a traceable numeric code, was authorized by the Careggi Transfusion Unit.

## 3. Results

### 3.1. Pre-Adaptation of E. coli to Tomato Pericarp

**Figure 1:**
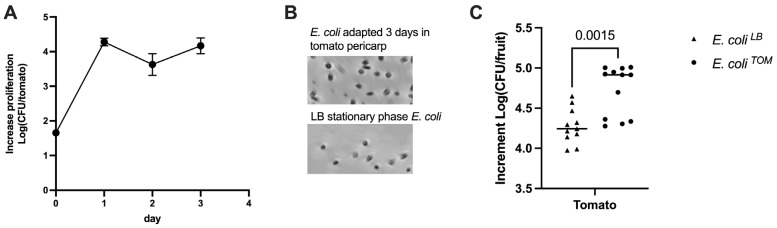
*E. coli* growth in tomatoes. (**A**) Increase in *E. coli* proliferation in tomato pericarp within 4 days. The decline of growth on day 2 is not significantly different in comparison to days 1 and 3 (*p* < 0.1650). Significance occurs only in comparison with day 0. (**B**) Micrograph showing *E. coli* ^TOM^ and *E. coli* ^LB^ cells (magnification of 400X). (**C**) Growth of pre-adapted *E. coli* ^TOM^ and *E. coli* ^LB^ in tomato pericarp. Error bars represent standard error. Horizontal bars in (**C**) show the significance of the T-test (*p* < 0.05).

About 150 cells of *E. coli* ATCC 35218 were inoculated in three shallow wounds in the tomato pericarp, showing a growth of about 2 Log(CFU/tomato) within only 24 h (Figure 1A) and reaching the stationary phase within two days.

We further determined whether pre-adaptation of *E. coli* in tomato pericarp (*E. coli* ^TOM^) would support an increase in proliferation if transferred to other vegetables. Therefore, other tomato fruits were contaminated with the *E. coli* ^TOM^ strain and the *E. coli* originating from a stationary LB culture (*E. coli* ^LB^) (no evident differences in cell morphology were observed in these two bacterial populations used for fruit contamination, Figure 1B). It is reasonable to assume that the pre-adaptation of *E. coli* ^TOM^ would be advantageous, growing at a higher rate when transferred into a new vegetable when compared to *E. coli* ^LB^. This hypothesis was confirmed, showing that *E. coli* ^TOM^ had an increased—although limited—proliferation in tomatoes of log 4.7 CFU/fruit versus the log 4.2 CFU/fruit of *E. coli* ^LB^ (Figure 1C). Thus, pre-adaption positively affected *E. coli* fitness even when new vegetables were contaminated.

### 3.2. Differential Methylation Analysis and Expression of Genes Involved in Bacterial Adaptation

**Figure 2:**
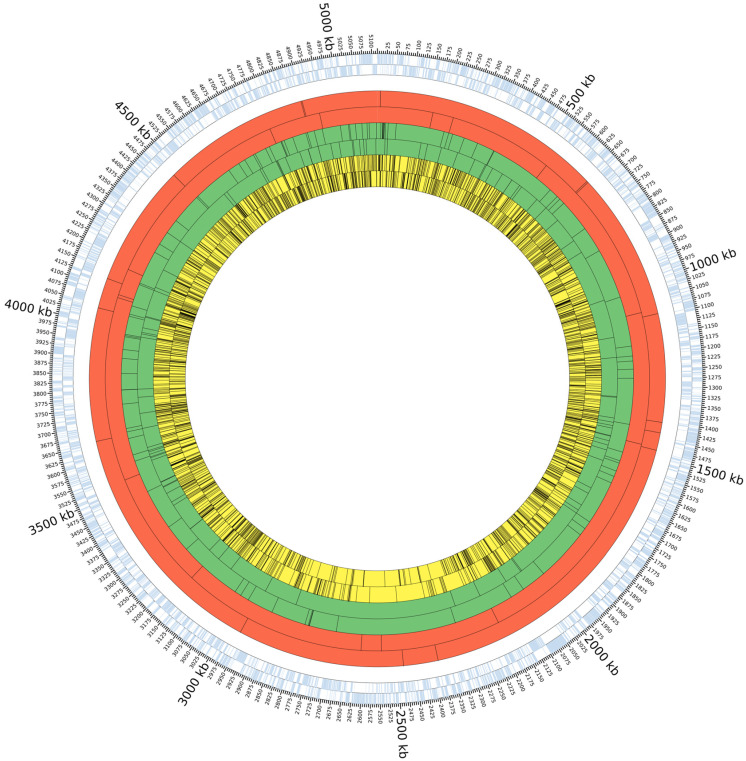
Circos plot of the results of the methylation analysis. Starting from the outside, plotted tracks represent the location of *E. coli* ATCC 35218 CDSs (light blue); the methylation status of A in the *dam* patterns (red); the methylation status of C in the *dcm* patterns (green); and the methylation status of C in CpG patterns (yellow). For methylation sectors, the direction (up/down) of bars indicates the strand where methylation has been called.

These results suggested that pre-adaptation in tomatoes probably influenced the expression of genes involved in interaction with the host environment. To further investigate this hypothesis, high-throughput Nanopore sequencing was performed to identify genomic loci whose expression could be affected by epigenetic modifications. Therefore, to identify genes putatively involved in the adaptation of *E. coli* ATCC 35218 to tomato pericarp, differential cytosine methylation was analyzed by sequencing the DNA of *E. coli* ^TOM^ and *E. coli* ^LB^. We selected a few significantly differentially methylated regions associated with a number of ORFs (open reading frames) (Figure 2, Table 1).

The criteria for choosing genes characterized by differentially methylated cytosines are described in the Materials and Methods section. Thus, *narG*, *papA_1*, *fimH*, *atoBCD*, *acrF*, and *murE-ftsI-ftsL* were selected for further investigations (Table 1).

All selected genes are involved in cell adhesion and resistance against toxic compounds: their expression was therefore studied via qPCR to quantify differences between the two conditions and to find a preliminary relationship between expression levels and methylation profiles. Almost all selected genes were more expressed in *E. coli* ^LB^, except for *acrF,* which was slightly more expressed in *E. coli* ^TOM^ (Table 1).

### 3.3. Effects of Pre-Adaptation on Environmental Stress Resistance and Cell Motility

**Figure 3:**
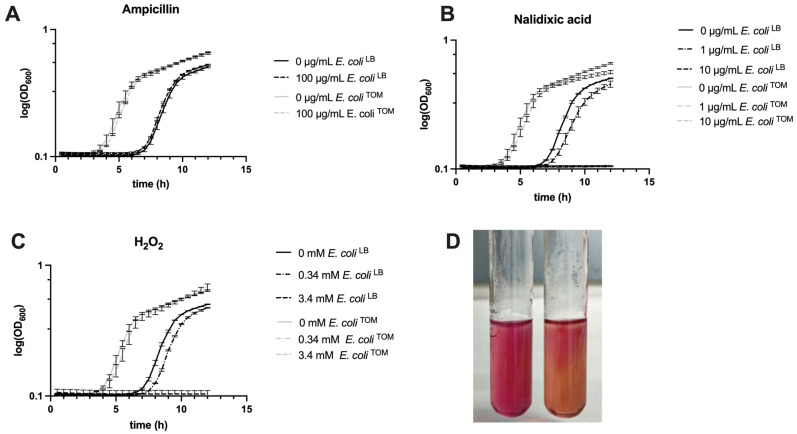
Different growth of *E. coli* ^TOM^ and *E. coli* ^LB^ in different conditions. Growth curves of *E. coli* ^TOM^ and *E. coli* ^LB^ in the presence/absence of (**A**) ampicillin (control), (**B**) nalidixic acid, and (**C**) H_2_O_2_; (**D**) different acidification by *E. coli* ^TOM^ (left tube) and *E. coli* ^LB^ (right tube) into semi-solid MacConkey, possibly indicating different motility. Error bars represent standard error.

We further focused on *acrF* because it encodes for a multidrug export protein and is involved in drug extrusion [24]. Thus, *E. coli* ^TOM^ and *E. coli* ^LB^ were cultivated in the presence of two antibiotics (i.e., ampicillin and nalidixic acid) and H_2_O_2_. Ampicillin was used as a control because *E. coli* ATCC 35218 carries a resistance gene, while H_2_O_2_ was tested because it is present in tomato wounds. Growth curves of *E. coli* ^TOM^ and *E. coli* ^LB^ growing in the presence of these compounds were compared and are shown in Figure 3.

In all experiments, the pre-adapted *E. coli* ^TOM^ showed a reduced lag phase, entering the exponential phase about 4 h earlier than *E. coli* ^LB^. In addition, *E. coli* ^LB^, which had a lower expression of *acrF*, was more sensitive to 1 µg/mL nalidixic acid and 0.34 mM H_2_O_2_ when compared to *E. coli* ^TOM^, as represented by the further delay of the curves of *E. coli* ^LB^.

We, therefore, focused our attention on two other particular genes: *papA_1* and *fimH,* which are remarkably less expressed in *E. coli* ^TOM^ and encode for components of fimbriae. In the literature, it is reported that the decrement of fimbriae could be associated with reduced cell motility [25]. Hence, a motility test was performed, supporting the reduced motility of E. coli ^TOM^ compared to *E. coli* ^LB^ (Figure 3D).

### 3.4. Effects of Pre-Adaptation on Pathogenicity

**Figure 4:**
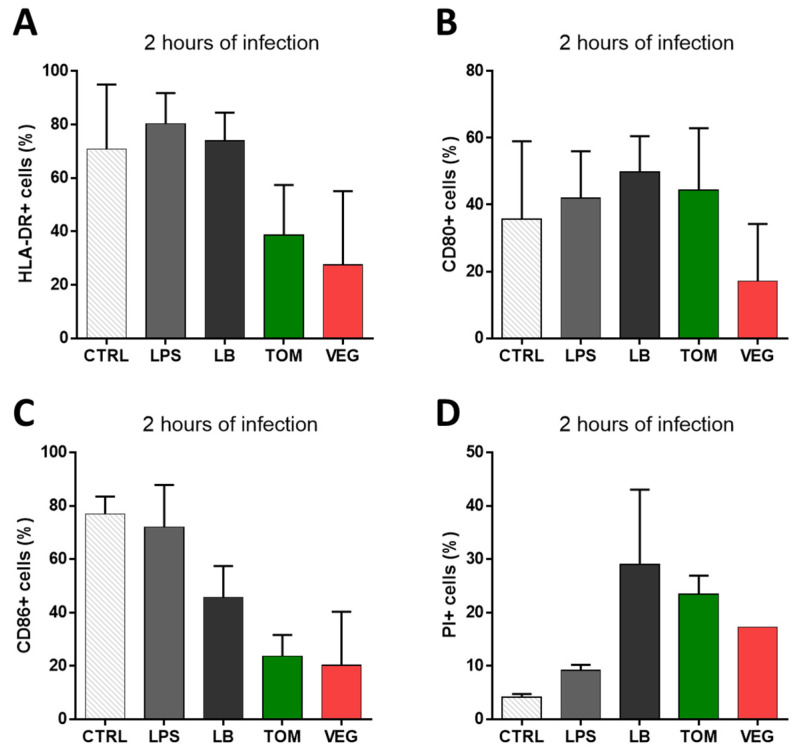
Activation and viability profile of mDCs after in vitro infection with *E. coli* analyzed by flow cytometry. Histograms represent the percentage of mDCs cells positive for (**A**) HLA-DR, (**B**) CD80, (**C**) CD86, and (**D**) propidium iodide (PI) after being cultured under different conditions: without any stimulus (control, CTRL), in the presence of only LPS (LPS), with *E. coli* ^LB^ (LB), with *E. coli* ^TOM^ (TOM), or with non-contaminated tomato (VEG). Data were analyzed using GraphPad Prism v.6 software, with one-way ANOVA test followed by Tukey’s post hoc test for multiple comparisons. Error bars represent the standard error of the mean.

Finally, we measured to what extent the pre-adaptation of *E. coli* ^TOM^ induced any inflammation response in human mDCs, as a result of adaptation to the host environment. Indeed, previous studies showed that commensal *E. coli* could elicit strain-specific inflammatory stimuli [18]. *E. coli* ^TOM^, *E. coli* ^LB^, and non-contaminated tomatoes (used as control) were tested on mDCs. The activation status and viability of mDCs after 2 h of in vitro infection with different treatments were evaluated by flow cytometry, analyzing the human leukocyte antigen molecule HLA-DR (HLA) (Figure 4A), analyzing the expression of surface markers CD80 and CD86 (Figure 4B and Figure 4C, respectively), and by assessing the percentage of PI+ cells (necrotic cells) (Figure 4D). CD80 and CD86 are costimulatory molecules necessary for activating naïve T lymphocytes and are typically upregulated on activated mDCs. HLA-DR, also expressed on activated mDCs, is a class II HLA molecule essential for antigen presentation to CD4+ T helper lymphocytes (Th).

The results showed a trend towards the reduced expression of HLA-DR and CD86 in mDCs infected with either *E. coli* ^TOM^ or non-contaminated tomato (Figure 4).

A similar trend was observed with a reduction in the percentage of CD80+ cells in the presence of non-contaminated tomatoes. Regarding viability, the percentage of necrotic PI+ cells tended to increase compared to the controls (i.e., non-infected mDCs and mDCs grown in the presence of LPS), reaching about 20–30% in case of infection with *E. coli* ^TOM^ and *E. coli* ^LB^. However, these differences were not found to be statistically significant.

## 4. Discussion

This study aimed to investigate unexplored aspects of the interaction between bacteria and plant hosts, analyzing pre-adaptation effects in an *Escherichia coli* grown in a fruit endocarp. The underpinning hypothesis is that pre-adaptation may increase bacterial virulence, providing an “epigenetic memory” that would foster replication in vegetables and other hosts such as humans [26].

In this regard, this proof-of-concept study conducted with a non-pathogenic *E. coli* strain provided indications for future directions. *E. coli* pre-adapted in tomatoes showed a higher fitness during a second round of fruit contamination, reaching higher titers compared to non-adapted bacteria. Additional studies should be performed in order to further confirm this observation, since although the difference is significant, it is also limited to 0.5 log in tomato. Similar behavior was observed previously in the case of lettuce, where *E. coli* K12 isolated from colonized leaves progressively colonized lettuce seedlings to a greater extent [11].

Bacteria face a challenging environment when they enter plant tissues, which are rich in both nutrients and stressors. Indeed, depending on the pathogen-associated molecular patterns (PAMP) recognized by the plant, the host may activate the production of reactive oxygen species (ROS) in the intercellular space. For example, tomato exhibits microbursts of H_2_O_2_ when facing particular stressors, including invading pathogens [27,28]. Thus, in the context of this pathogen–host competition, it should not surprise that other studies demonstrated that plant-adapted *Escherichia coli* showed increased resistance to oxidative stress. It is reasonable to assume that several factors are regulated in bacteria facing such a stressful environment, such as those involved in osmoprotection, as reported in the case of *E. coli* colonizing lettuce [29], and critical de novo biosynthesis of amino acids, as in the case of *Salmonella* [30]. Conversely, fruits are also rich in nutrients, vitamins, and other essential elements that sustain invasive microbes, leading to their adaption and proliferation. During ripening, the host’s nutrients may modify signaling mechanisms in the pathogen leading to metabolic responses and modification in the synthesis of pH-modulating molecules and in carbon-regulation signaling [31]. These changes occur even though *E. coli* and its closest taxonomical enterobacteria are commensal rather than plant pathogens; thus, this aspect requires further investigation.

Identification of genes potentially activated/repressed in *E. coli* upon invasion of tomato pericarp was investigated through a differential methylome analysis. This approach allowed us to identify six differentially expressed genes (i.e., *narG*, *papA_1*, *fimH*, *atoBCD*, *acrF*, and *murE-ftsI-ftsL*). All of them were more expressed in *E. coli* ^LB^ except for *acrF*, which was more expressed in *E. coli* ^TOM^. The higher expression of *acrF* could be related to the increased resistance of *E. coli* ^TOM^ to harmful compounds, as demonstrated here with ampicillin, nalidixic acid, and H_2_O_2_, compared to not pre-adapted cells. This finding is in agreement with previous experiments showing a dramatic increase in tolerance to H_2_O_2_ in plant-adapted *E. coli* K12 cells [11]. However, it cannot a priori be excluded that the increased resistance of *E. coli* ^TOM^ could be due to the expression of other genes (e.g., not necessarily those encoding for efflux pumps) and activation of additional detoxification pathways (e.g., genes involved in oxidative stress tolerance), as it was demonstrated by microarray-based whole-genome transcriptional profiling in *E. coli* harvested from lettuce [11,32].

Finally, we tested to what extent the adaptation of *E. coli* ^TOM^ was able to modify the inflammation response in human mDCs. We did not find significant differences in the activation and viability profile of mDCs after in vitro infection with adapted *E. coli* when compared with the non-adapted one. However, new experiments should be further conducted: for example, an improved method to isolate *E. coli* from the tomato pericarp should be established since cell viability was compromised even in the case of non-contaminated tomatoes used as control.

## 5. Conclusions

In conclusion, the findings reported in this study help to understand the physiology of *E. coli* during the adaptation process following the colonization of tomato pericarps. It would be relevant to repeat these experiments with human pathogens and those more related to the food industry (e.g., *Salmonella* and pathogenic *E. coli*). Furthermore, it would be interesting to couple the characterization of the methylome with a transcriptomic analysis: this approach would allow a more comprehensive genome-wide overview of the bacterial adaptation to the fruit tissues. Previous research has shown that *Salmonella* replicates differently in relation to the ripening stage (i.e., green, partially ripe, or ripe) [33,34]. This different stage-dependent behavior should also be analyzed in the case of *E. coli* to better understand to what extent each ripening stage affects *E. coli* methylome and gene expression.

The biology of the interaction between *E. coli* and tomatoes should be further studied to obtain more insights into more practical aspects (as has already been performed in the case of *Salmonella*), such as the effect of humidity on contamination in the field [6], how agronomical practices can prevent *E. coli* contamination of fruits [35], the possible use of signaling molecules to reduce enteric contamination [36,37], and the selection of *cultivars* less susceptible to enteric pathogens [38].

Understanding the physiology of human pathogens in vegetables would allow us to better understand their life cycle outside the animal hosts and prevent food contamination.

## Figures and Tables

**Table 1:** Cytosine methylation state of *E. coli* ^TOM^ and relative gene expression in tomato pericarp.

Gene	Function	Methylation ^1^ in *E. coli* ^TOM^	Relative Expression (*E. coli* ^TOM^ vs. *E. coli* ^LB^)
*narG*	Respiratory nitrate reductase 1 alpha chain	+	−13.9
*papA_1*	Pap fimbrial major pilin protein	−	−11.5
*fimH*	Type 1 fimbrin D-mannose specific adhesin	−	−36.1
*atoBCD*	Metabolism of Acetyl-CoA	+	−2.52
*acrF*	Multidrug export protein AcrF	−	+1.6
*murE-ftsI-ftsL*	Peptidoglycan	+	−1.3

^1^ Sites significantly more (+) or less (−) methylated in adapted *E. coli* ^TOM^ in comparison to *E. coli* ^LB^.

## Data Availability

The data presented in this study are available on request from the corresponding author.

## Supplementary Materials

The following supporting information can be downloaded at https://www.mdpi.com/article/10.3390/biology12040633/s1. Table S1: Primers used in this study.
