# Supplementary material for: Adaptation of Commensal Escherichia coli in Tomato Fruits: Motility, Stress, Virulence
Source: Biology (Basel). 2023 Apr 21;12(4):633. doi: 10.3390/biology12040633 (PMC10136321; doi:10.3390/biology12040633)
Supplement: Supplementary file 1 [file biology-12-00633-s001.zip › biology-2286071-supplementary.pdf]

Supplementary material - Table S1. Primers used in this study.

Gene	Function	Forward 5'-3'	Reverse 5'-3'
narG	Respiratory nitrate reductase 1 alpha chain	TCTATAACCACTCCAGCCAG	CTCTTTCAGGGATTTCACCG
papA_1	Pap fimbrial major pilin protein	AGTCTATTGATTTTGGACAACTTTC	GGCAGTAATGTCACAATTAACTAAT
fimH	Type 1 fimbrin D-mannose specific adhesin	CGGGTTGTTTATAATTCGAGAAC	ATCATTATTGGCGTAAATATTCCAC
atoB-C-D	metabolismo AcelitCoA	CTGCTCCTCGACGAAATTGG	TGCCTTCTTTCACCATTGCC
acrf	Multidrug export protein AcrF	GGGTGCTGGCCATTATTCTG	GTTCGATAACCTGCGTCACC
murE-ftsl-ftsL	Peptidoglycan	GGGCGATGGGGAAATTGAAA	GAACACTTCCATACGTCCGC
fliC	flagellin	ATAATCTACGCCGCCAACT	GACTCCATCCAGGACGAAA
flHD	Gene Involved in biofilm formation	ATCGTCTGGTGGCTGTCAA	GTCCGCTATGTTTCGTCTCG
16S	Ribosomal DNA	CCTACGGGNGGCWGCAG	GACTACNVGGGTWTCTAATCC
rpoD	RNA polymerase σ^{70} factor	AGCAAAACCCGCAGTCAC	CCATGTCGTTGATCATTTGG
